# Our Correspondence

**Published:** 1870-01

**Authors:** 


### Our Correspondence.

WELLSBORO', Pa., Nov. 10, 1869.

To the Editor of the BISTOURY:

In the article "The Stomach Æsthetically considered," in your October number, your correspondent, or rather your contributor ISHMAEL makes a grave error of confounding the words stomach and belly as synonyms of each other. He charges Clement Moore with "ignorantly" using the word belly to designate the rotund stomach of Santa Clans;—Washington Irving (Deidrich Knickerbocker) with introducing it "tenderly and affectionately in his description of the "personal appearance of the waddling heroes of the New Netherlands;" and Shakespeare with "alluding" to it in one or two instances. Now, I deny that either of the authors mentioned used the word in question as synonymous with the word stomach, and I affirm that each of them in using it intended to mean the front part of the human trunk—the entire viscera—which, of course, includes the stomach and the other organs which make up the rotundity of the "human form divine."

A word about the word "belly." Ishmael says it is an "unpleasant" word. I do not think so. It is a much more euphonious word than stomach. I know it is considered vulgar to use it in "good society," but the best writers have used it in the sense spoken of. When the bible was translated into English it must have been in common use in "our best society," for in that book it is used as a synonym of the bowels, the stomach, the womb, the carnal heart, the whole man, &c.

Ishmael is considered a good writer in this latitude, and, as he is doubtless regarded as a model, he should be careful not to lead younger writers into the error of confounding the whole with any one of its parts.

Yours, PATRICK JAMES M'WHIRTER.

SYRACUSE, N. Y. October 20th, 1869.

DEAR BISTOURY.

You are a real health promoting, companionable, lively little friend that it is a pleasure to receive in one's family. We look for your coming anxiously and the only fault we can possibly find with you is that you don't come often enough. Now BISTOURY, you seem to love babies, if I may judge from the many valuable and funny articles you have given us in relation to them. You also advocate the raising of large families and sensure severely, infanticide. That is all right—I agree with you. But dear BISTORY what are we poor sickly, feeble wives to do who are made miserable by *excessive* child bearing? Can I, who have given birth to a child regularly, once every year since marriage, and who have been made feeble and sickly in consequence of it—can I bear healthly and vigorous children, such as you so often picture to us? O, if you knew the torture that many of us poor wives suffer from inconsiderate, healthy, robust husbands, who compel us to satisfy their animal passion and thus force us to give life to children that we desire not; I am sure you would pity us and offer us, or our husbands, some advice upon the abuse of the marriage relation.

Believe me, BISTOURY, I do desire to be a faithful, submissive wife and mother; but is there not some way by which us poor wives can be all this, and not be rendered miserable for life? Is there no means by which we can be *dutiful* wives yet have control of our own persons?

Patiently Yours,

A WIFE AND MOTHER.

We wish that the letter of this poor, abused wife, might reach the eye and consciousness of every married man in the land. For we fear there exists very few men who do little else than render the marriage relation a species of legal prostitution. The marriage rite was not created with a view of affording man an opportunity for gratifying his lust, as many are wont to suppose. Yet, men accept this interpretation and absolutely outrage the persons of their helpless and once loving wives. We firmly believe that more poor women are carried to their graves, martyrs to their husband's passions, than from all other causes combined.

We know, from our experience as a physician, that *nearly all* so-called "female weaknesses" are the direct result of sexual abuses.

We know of no remedy for this terrible evil except to have more intelligent health journals in the land, and to strive to gain a place for them in every family. While every man subscribes for his newspaper, let every woman secure a health journal in her family, and when such articles as this find their way into its columns, let the wife present it to the husband for perusal, and if he loves her, and is not a brute, he will certainly have sufficient respect for his wife and moral decency, not to make her the victim of his unholy passion. When, as is too often the case, the child is begotten *by accident* and the mother weeps and deplores the fate that *compels* her to motherhood, while the prospective father is sullen and cheerless over the same fate—but gratifying his lust the same during the development of the child—is it any wonder that so many sickly, puny, little ones grow up among us?

We believe it to be healthful, noble, and the *duty* of every healthy married woman to become the mother of from two to four children; and these children should be begotten *purposely* and during the period of the best health of both husband and wife. We also believe and *know* it to be the duty of every husband to be careful of his wife's health and person, and not convert a fond and patient wife into a *mistress* for the gratification of his lust.

---

YPSILANTI, 14th Oct., 1869.

Thank you, my dear Bistoury, glad to see you again. 'Pon my honour welcome again, I am disposed to censure you for tardiness but in consideration of your voluntary appearance for a year or thereabouts, I refrain from scolding you. Now Bistoury, my pleasure giving companion, perhaps you do not know, that on the 26th day of April, 1869, I sent Fifty Cents (lawful shinplaster) to one UpDeGraff of Elmira, with earnest request that he should get a ticket for you and let you come directly, constantly, aud periodically,

To

J. W. B., M. D.,
YPSILANTI, Michigan.

The money was received Doctor and placed to your credit. Hope BISTOURY will reach you regularly and give you as much pleasure to read as it does us to edit.

---

TROY, Pa., Nov. 1, 1869.

EDITOR BISTOURY,

*Dear Sir:*—I see you are exposing a great many humbugs throughout the country, fellows who advertise to cure disease by sending medicine and formula, so I thought I would drop you a line and tell you how *I* have been victimized.

I saw in a New York paper that Sayre & Co. would send a sample of their great remedy for consumption, *free* upon application. I sent—I got some—I took it— it was a red-looking bitter powder—it did me no good of course. While I was debating whether I should send for more and give it a "fair trial," I received a lithographed letter from Sayre & Co., with my name written in same style of chirography as the body of letter, setting forth that they were sure the medicine would do me good and that I had better send them $3,00 for a sample box of the medicine, as it had cured hundreds, &c.

This letter first made me suspicious, as it was intended to convey the impression that it was purposely written for *me*, still I sent the money and received the package of medicine, with another letter directing me to send for more when the first had been taken, as from three to ten packages were often necessary to effect a cure. Now, I have expended thirty dollars in this trash and I am worse off than before, for I might have been taking medicine the while, that would have done me good. * * *

I have returned to my family physician, as pr. advice from your BISTOURY, and have concluded not to invest any more money with Sayre & Co., or with that other notorious humbug, *Rev.* (?) Edward A. Wilson.

J. R. M.

A sensible conclusion to come to. We hope others similiarly afflicted will profit by J. R. M's experience and let every miserable, advertising, swindling humbug alone.

---

SALAMANCA, N. Y., Nov. 6, 1869.

THAD. S. UP DE GRAFF, M.D.,

*Dear Sir:*—Here is another contribution to the long list of swindlers you have already exposed in your BISTOURY:—My mother, who was a stout healthy woman of sixty-five years of age, had a small, red looking spot upon one of her breasts. It annoyed her very much, as she was afraid it was a cancer, yet it gave her no pain whatever. Last summer a travelling "cancer doctor" came to our town and advertised to cure all kinds of cancers. Mother was induced to visit him and show him her supposed cancer. He, without hesitation, pronounced it a "spider cancer," and that it would soon be past cure unless she employed him to remove it. Contrary to my wishes and the advice of our family physician (who pronounced it harmless), she had a cancer plaster applied. It inflamed her breast so much within two hours that she became frightened and removed it. The doctor (?) came, however, and re-applied it, directing her to keep it on all night at all hazards. She did so. In the morning the breast was intensely inflamed, and before night mother was completely prostrated with pain and fever. In one week the breast ulcerated—soon red looking lumps began growing from it. This the doctor (?) called *proud flesh*, and said it would "all come off right." He applied caustics, but still it grew, and finally the chap decamped, saying that mother had "put it off too long," that it could not now be cured.

Mother suffered intensely for two months, and finally died in great agony, a victim of her own folly.

I write this as a warning to all persons similiarly afflicted, not to trust their lives in the hands of such miserable, blundering, travelling doctors. Hoping this may have a salutary effect upon your many readers,

I am, very truly yours,

R———

We have frequently cautioned our readers not to use "cancer plasters" as prescribed by so-called cancer doctors. We have seen many harmless moles and other excrescences upon the skin, converted into malignant sores by the use of such caustics. Never trust one who comes to your door in search of cancers—he is not the sort of man that can cure them.

---

JACKSONVILLE, Fla., Oct. 20, 1869.

DR. THAD. S. UP DE GRAFF,
EDITOR BISTOURY.

*Sir:*—I saw a copy of your journal here today and read the letters about those men who send medicine for "seminal weakness." I have been patronising them for two years, and had no idea that I was being cheated until I read those letters. I have paid a fellow that calls himself John B. Ogden, in New York, about one hundred dollars for medicines that have done me no good—but he kept promising to cure me all the while, and I did not know where else to apply. Why don't you tell us unfortunates where to go for relief?

J. T.

There are skillful physicians in almost every city who can prescribe for your case successfully and understandingly. They are *not* the men, however, who advertise to send you *free* formulæ, or who publish such trash as—"Manhood how lost, how restored," Marriage Guides," etc., etc.

---

The following encouraging note, from a professional brother speaks for itself:—

HISEVILLE, Kentucky, Nov. 3, 1869.

DR. T. S. UP DE GRAFF,
Elmira, N. Y.,

*Dear Sir:*—Inclosed please find one dollar, for which please send two numbers of your "Bistoury" for one year, to———. I have been taking your very valuable journal, "The Bistoury" for nearly two years, and have sent you a good many names, as subscribers, and all of whom are very much pleased with it, so much so that they now say that they cannot do without it. I am well satisfied that it is doing a great work for the medical profession, by its grand exposition of charlatanism, and the introduction of the true principles of medical and surgical science to the entire reading people. Long may you live and prosper in your enterprise.

Your Friend, and Brother,
T. L. N., M. D.

---

OPIUM SMOKING.—The report for 1867, from the Bureau of Statistics of the United States Treasury Department, contains the surprising statement that, during the year 50,551 pounds of opium, *prepared for smoking*, were imported into this country. Doubtless most of it was consumed by our Chinese friends.

---

WISCONSIN has a boy, aged fourteen years, who weighs but eighteen pound, and another, eleven years of age, weighing 135 pounds. Who wants to start a show?

